# Disseminated fusariosis with endophthalmitis in a patient with hematologic malignancy

**DOI:** 10.1590/S1679-45082013000400026

**Published:** 2013

**Authors:** Guilherme Fleury Perini, Luis Fernando Aranha Camargo, Claudio Luiz Lottenberg, Nelson Hamerschlak

**Affiliations:** 1 Hospital Israelita Albert Einstein, São Paulo, SP, Brazil

A 68-year-old patient previously diagnosed with acute myeloid leukemia had fever and myalgia during chemotherapy. Despite broad spectrum antibiotics, fever persisted and, after 3 days, skin lesions compatible with *Fusarium* infection were seen on patients' lower limbs. Dyspnea and hypoxia were observed, and computerized tomography showed extensive pulmonary infiltrates; blood cultures were positive for *Fusarium* sp. A diagnosis of disseminated fusariosis was done, and lipossomal amphotericin, voriconazole and granulocyte infusion were initiated.

**Figure 1:**
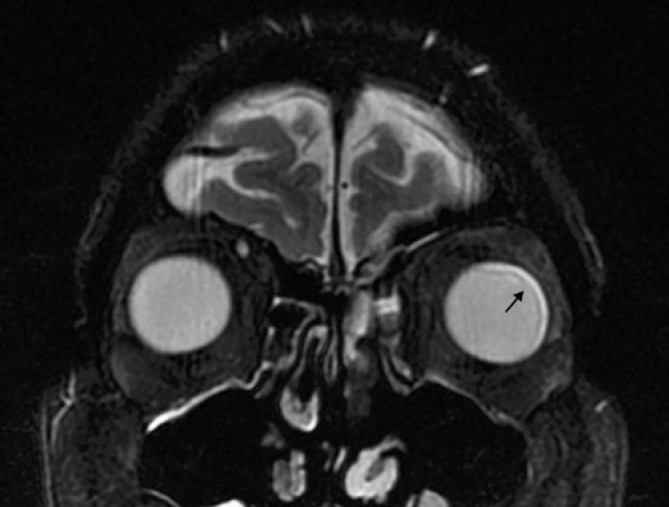
Orbit computerized tomography scan showing anterior, medial and lateral delamination of left eye, which was compatible with endophthalmitis

**Figure 2:**
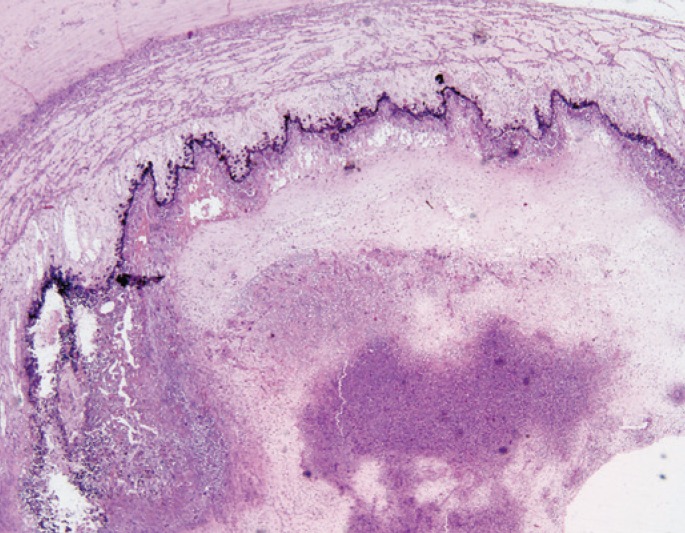
Anatomopathological exam of left eye showing intraocular abscess

**Figure 3:**
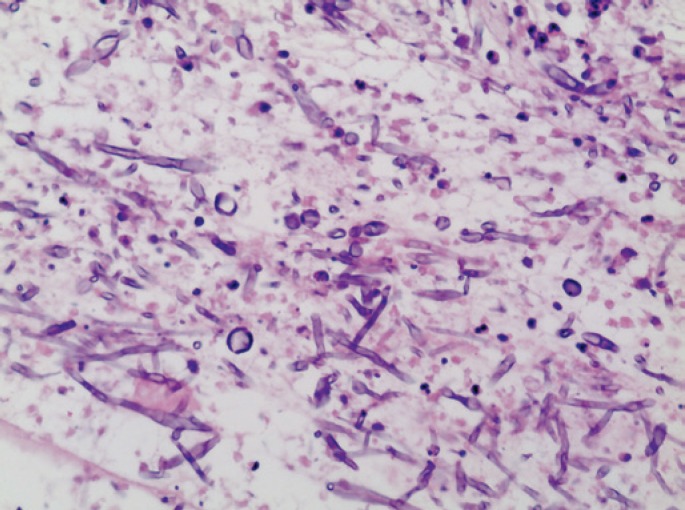
In a higher magnification, *Fusarium* sp hyphae can be seen

The patient had complete regression of skin lesions and pulmonary infiltrates, but a week later he complained of visual blurring in the left eye. An orbital magnetic resonance imaging showed enhancement of left ocular globe with a lateral, medial and anterior delamination that was compatible with endophthalmitis (Figure 1). An intraocular treatment with voriconazole was applied and a little improvement was seen. *Fusarium* sp endophthalmitis affecting his right eye was diagnosed, which justified his visual loss. Despite treatment, a progressive worsening of bilateral endophthalmitis occurred and, to control the disease, the eye was enucleated. The pathological examination of the enucleated eye showed an intraocular abscess adjacent to the retina (Figure 2). In a higher magnification, *Fusarium* hyphae could be identified (Figure 3).

*Fusarium* species cause a broad spectrum of infections in humans, including superficial, locally invasive, and disseminated infections. Immunocompromised patients are at higher risk, especially those with prolonged and severe neutropenia and/or severe T-cell immunodeficiency^(1)^. Patients with acute leukemia and patients undergoing hematopoetic stem cell transplantation are particularly at risk, especially to the invasive and disseminated^(2)^ forms. The typical pattern of disseminated disease is a combination of cutaneous lesions (often with external necrosis in the center of the lesion), positive blood cultures, and with or without involvement at other sites (sinuses, lungs, and others)^(3)^.

*Fusarium* endophthalmitis in the immunocompromised host usually results from hematogenous seeding^(4,5)^. Intraocular and systemic therapies often have poor responses. In order to avoid central nervous system involvement, the evisceration of the eye may be necessary^(6)^. Few case reports describe successful treatment of *Fusarium* sp endophthalmitis with voriconazole alone or in combination with caspofungin and posaconazole^(7,8)^.
